# Impact of implementing a pediatric early warning system on outcomes in hematopoietic stem cell transplant units in South America and Europe

**DOI:** 10.3389/fonc.2025.1712611

**Published:** 2025-12-09

**Authors:** Monica L. Quijano-Lievano, Oscar Ramirez, Yichen Chen, Maricela Robles-Murguia, Meenakshi Devidas, Hilmarie Muñiz-Talavera, Adolfo Cárdenas-Aguirre, Carlos Portilla, Diana Castrillon, Diana Rendon, Andreia Ribeiro Pereira Aguiar De Paula, Rosdali Diaz-Coronado, María Sánchez-Martín, Silvio Torres, Verónica Soto Chávez, Asya Agulnik

**Affiliations:** 1 Health Doctorate Department, Universidad del Valle, Cali, Colombia; 2 Bone Marrow Transplant Unit, Clínica Imbanaco, Cali, Colombia; 3 Cali Population-Based Cancer Registry, Department of Pathology, Universidad del Valle, Cali, Colombia; 4 Research Unit, Fundación Pediatric Oncologists-Hematologists (POHEMA), Cali, Colombia; 5 St. Jude Children’s Research Hospital, Memphis, TN, United States; 6 Pediatric Department, Medicine School, Universidad del Valle, Cali, Colombia; 7 Pediatric Hemato-Oncology Unit, Clínica Imbanaco, Cali, Colombia; 8 Hospital do Amor/Hospital de Cancer Infanto Juvenil de Barretos, Barretos, Brazil; 9 Instituto Nacional de Enfermedades Neoplásicas (INEN), Lima, Peru; 10 Hospital Universitario La Paz, Madrid, Spain; 11 Doctorate School, Medicine Faculty, Universidad Autónoma de Madrid, Madrid, Spain; 12 Hospital Universitario Austral, Buenos Aires, Argentina; 13 Hospital Civil de Guadalajara, Guadalajara, Mexico

**Keywords:** pediatrics, cancer, hematopoietic stem cell transplantation, early warning score, critical illness, pediatric intensive care unit

## Abstract

**Background/Aim:**

Escala de Valoración de Alerta Temprana (EVAT) is a Spanish-language Pediatric Early Warning Score (PEWS) validated to predict the need for unplanned intensive care unit (ICU) transfer in hospitalized children with cancer. We assess the effect of PEWS implementation on hospitalized children undergoing hematopoietic stem cell transplantation (HSCT) in variable-resource centers in South America and Europe.

**Methods:**

We analyzed data from a prospective registry of clinical deterioration events (CDE), defined as an unplanned ICU transfer, ICU-level intervention on the ward, ward cardiopulmonary arrest, or deterioration to death, among hospitals implementing PEWS. We included CDE occurring in patients under age 19 hospitalized in an HSCT unit between April 2017 and June 2024. We used the Wilcoxon rank sum test, chi-square test, and Fisher’s exact test to compare CDE occurring before PEWS implementation (pre-PEWS) and during/after PEWS implementation (post-PEWS).

**Results:**

During the study period, we identified 221 CDE among 146 patients from six centers (South America *n =* 5; Europe *n =* 1). The median age was 9 years (IQR, 4–14), 68% (*n =* 150/220) were male, and 20% (*n =* 44/221) experienced more than one CDE. The overall event mortality rate was 18% (*n =* 39/221). Of 221 CDE, 71 occurred pre-PEWS implementation and 150 post-PEWS implementation. CDE post-PEWS were less likely due to respiratory distress (38%, *n =* 27/71 *vs*. 23% *n =* 35/150, *p =* 0.0348), had fewer organ systems with dysfunction at ICU transfer (median 2 [IQR 1–3] *vs*. 0 [IQR 0–2], *p < 0.*001), and had lower PIM2, which indicates a lower severity of illness at ICU transfer (median 5.0 [IQR 1.4–7.9] *vs*. 1.5 [IQR 1.2–3.4], *p <* 0.001). There was a non-significant decrease in clinical deterioration event mortality post-PEWS implementation (24%, *n =* 17/71 *vs*. 15%, *n =* 22/150, *p =* 0.1335).

**Conclusions:**

PEWS implementation promoted the early identification of critical illness as well as ICU transfer at a lower severity of illness for children undergoing HSCT. This work further supports the use of PEWS in the care of all children with cancer and blood disorders globally.

## 1 Introduction

Hospitalized pediatric patients diagnosed with cancer and those undergoing hematopoietic stem cell transplantation (HSCT) are at a high risk for clinical deterioration and death, especially in resource-limited settings. Pediatric early warning score (PEWS) systems aid in the early identification of clinical deterioration in hospitalized children (1–6). The system includes a scoring scale that enables nursing staff to assess hospitalized patients based on vital signs and clinical status as part of routine care. It also includes a response algorithm that guides healthcare team interventions for patients with clinical deterioration (5). Escala de Valoración de Alerta Temprana (EVAT) is a Spanish-language PEWS that has been validated to predict the need for unplanned transfer to the pediatric intensive care unit (PICU) in hospitalized children with cancer (2, 5). However, the study of its use in the care of children undergoing HSCT is limited.

Proyecto EVAT is a quality improvement collaborative initiated by St. Jude Children’s Research Hospital (7) in partnership with regional leaders in Latin America and Europe to improve outcomes for children with cancer who are at risk for deterioration (4, 8). The regional experience with Proyecto EVAT demonstrates a successful strategy for adoption and scale-up of an evidence-based practice in real-world settings (8), with more than 110 pediatric oncology centers implementing and sustaining PEWS. PEWS implementation through Proyecto EVAT has been shown to reduce clinical deterioration mortality in participating centers regardless of their resource level (4).

Prior studies suggest that early transfer to the ICU may lead to better outcomes for patients undergoing HSCT who develop critical illness (1–3, 5). The PEWS informing EVAT was validated in pediatric HSCT patients in the United States (3). However, information about how PEWS implementation affects clinical outcomes in this population in other settings is limited (9–12). This study evaluates the impact of PEWS implementation on hospitalized children and adolescents undergoing HSCT in variable-resource centers in South America and Europe.

## 2 Methods

### 2.1 Study design

We performed a secondary analysis of data collected for a prospective, multicenter cohort study of hospitals providing childhood cancer care in patients undergoing HSCT. Each of these hospitals participated in Proyecto EVAT, a Spanish-language PEWS adapted for low-resource settings (13). Registry methods have previously been described and are briefly summarized below.

### 2.2 Human subjects approval

The St. Jude Institutional Review Board reviewed this secondary registry-based de-identified data analysis as non-human subjects research. Every collaborating center received official local approval to carry out PEWS and take part in Proyecto EVAT. Where required, further institutional approvals were acquired following local regulations.

### 2.3 Setting

Of 96 centers participating in Proyecto EVAT as of June 2024, 12 centers could perform HSCT. Of these, six centers that had completed PEWS implementation and had both baseline and post-implementation registry data on clinical deterioration events (CDE) were included in this study. These six centers joined Proyecto EVAT after April 1, 2017 and completed PEWS implementation by December 31, 2023, allowing at least 6 months of post-PEWS implementation data collection. We define two periods: the period occurring before PEWS implementation (pre-PEWS) and during/after PEWS implementation (post-PEWS). In addition to facility data routinely collected by Proyecto EVAT (4), we surveyed each participating HSCT center on HSCT infrastructure (number of available pediatric HSCT beds, separation of the HSCT unit from the oncology ward, and ward nursing ratios) and transplant activity during the year of PEWS implementation (annual number and type of HSCT, CAR-T therapy, the underlying diseases transplanted, and intensity of conditioning).

### 2.4 PEWS

PEWS/EVAT uses a five-component rating system (neurological, cardiovascular, respiratory, and nursing and family concerns) based on vital signs, physical examination results, and treatment needs (**Supplementary Figure S1**). Hospitalized patients are rated by a bedside nurse after routine vital sign checks. This rating matches a clinical decision algorithm (**Supplementary Figure S2**) that indicates potential clinical deterioration and guides the clinical team toward appropriate treatment escalation.

### 2.5 Project PEWS/EVAT procedures

EVAT’s procedures have been described in detail previously (4). Briefly, Proyecto EVAT facilitates the implementation of PEWS in participating hospitals through a mentored implementation strategy (8). Hospitals join Proyecto EVAT through collaboration with St. Jude Global or by learning about the initiative from other collaborating institutions. Local teams who lead the PEWS implementation process consist of at least a ward nurse, ward physician, and critical care physician (4). The hospitals are guided through a three-phase process of planning, pilot implementation, and sustainability. Planning for implementation is the focus of the first phase (pre-PEWS); PEWS pilot and implementation are covered in the second phase (during PEWS); and PEWS sustainability is the focus of the third phase (post-PEWS). St. Jude and regional PEWS mentor center experts train and support local teams, helping them overcome barriers to adoption and ensuring the quality of PEWS implementation. Hospitals have completed implementation when they reach a specified level of PEWS quality, defined as having fewer than 15% PEWS errors for two consecutive months. Once implementation is complete, hospitals receive mentoring to create sustainability plans for the ongoing use of PEWS. Starting at the PEWS pilot and for 18 months post-implementation, participating centers collect data on the quality of PEWS use, including adherence to the PEWS algorithm, accuracy in calculating PEWS scores, and documentation of PEWS alongside vital signs. Weekly reviews of nursing records and monthly summaries are conducted to evaluate the use of PEWS among hospitalized pediatric cancer patients (5, 8).

### 2.6 Terminology

HSCT is a critical therapeutic intervention for pediatric patients with hematologic malignancies, immune deficiencies, and genetic disorders (8). The procedure involves infusing hematopoietic stem cells (along with hematopoietic progenitor cells) to reestablish the patient’s hematopoietic system. Generally, HSCT is performed after a preparative regimen consisting of agents designed to create marrow space, suppress the patient’s immune system to prevent rejection, and eradicate malignant cells in cancer patients (8).

Proyecto EVAT defines a CDE as any hospitalized patient who requires unplanned ICU transfer, receives an ICU-level intervention on the ward, including mechanical ventilation (invasive and non-invasive), vasoactive infusion, cardiopulmonary resuscitation (CPR), or ward cardiopulmonary arrest, or experiences a non-palliative death. A CDE ends at the moment of death, ICU discharge, or last ward-based ICU-level intervention (2, 4, 8). ICU-level interventions include CPR, vasoactive infusion, and invasive or noninvasive (continuous positive pressure and bi-level positive pressure) mechanical ventilation (4). ICU transfer is defined as the transfer to any hospital unit designed to provide a higher level than ward-based care to deteriorating patients (2). Clinical deterioration event-mortality is defined as a CDE that resulted in death in the ICU or within 24 hours of ICU discharge, or at the end of ward-based ICU interventions (14). The degree of illness severity and resource utilization is recorded at the start of the CDE (4). Deterioration in children with limitations on life-sustaining measures (do not resuscitate order or equivalent) was not considered a CDE and was not included in this study. Severity of illness and resource utilization describe the degree of critical illness at the start of the CDE (4). Sepsis and organ dysfunction are defined by the criteria proposed by Goldstein and colleagues (15). The Pediatric Index of Mortality 2 (PIM2) is calculated with standard criteria (16).

Ward cardiopulmonary arrest is defined as a clinical deterioration event requiring acute invasive mechanical ventilation or CPR or resulting in a non-palliative death on the ward (4). Ward CPR or death is defined as a CDE with cardiac arrest on the ward requiring CPR or resulting in a nonpalliative ward death (4, 15).

### 2.7 Measurements

Participating centers collected prospective data in a quality improvement registry from the start of participation in Proyecto EVAT through 18 months post-implementation. Variables gathered from clinical records included age, sex, neoplastic diagnosis, induction therapy for acute leukemia, and disease status. For each CDE reported, local site leads completed a de-identified case report form (**Supplementary Figure S3**) that included the following information: the cause of the CDE, the Pediatric Index of Mortality 2 (PIM2) severity score, physiological variables (neutrophil and platelet counts, lactate levels, and C-reactive protein), infectious variables (type of infection and any positive isolates), critical interventions (use of vasopressors, both invasive and non-invasive ventilation, renal replacement therapy, cardiopulmonary resuscitation), and transfer to a higher-level-of-care facility (ICU, intermediate care unit, emergency room) at the time of the CDE. We used hospital mortality and clinical deterioration events as the primary outcome variables. Additionally, the severity of illness on ICU transfer was described using the PIM-2 severity score and the presence of organ dysfunction at the time of transfer to a higher level of care. For each CDE reported, local site leads sent data to St. Jude for entry into a REDCap database (17). To ensure quality, clinical research associates at St. Jude regularly examined the data for missing or incorrect information.

### 2.8 Inclusion

The patients included in this study were under age 19 at the time of receiving the HSCT infusion, were hospitalized in the HSCT unit during the center’s quality improvement data collection period, and had data on deterioration events collected from the HSCT unit that had implemented EVAT/PEWS through Proyecto EVAT (**Supplementary Table S1**).

### 2.9 Statistical analysis

Descriptive statistics were used to define CDE characteristics among patients undergoing HSCT, pre-PEWS, and post-PEWS implementation at participating centers. The post-PEWS were collapsed in analysis due to the rapid uptake of PEWS use among these units and the similarities of data during these periods. We described hospital characteristics and HSCT services across centers (**Supplementary Table S2**). To describe the impact of PEWS on CDE, we compared events pre-PEWS and post-PEWS implementation. The CDE was used as the unit of analysis and CDE mortality as the primary outcome. Wilcoxon rank sum test, chi-square test, and Fisher’s exact test were used to assess event-level association between continuous/categorical CDE characteristics pre- and post-PEWS implementation. *P*-values <0.05 were considered statistically significant. Data were analyzed using R, version 4.4.1.

## 3 Results

### 3.1 HSCT characteristics of participating centers

**Figure 1:**
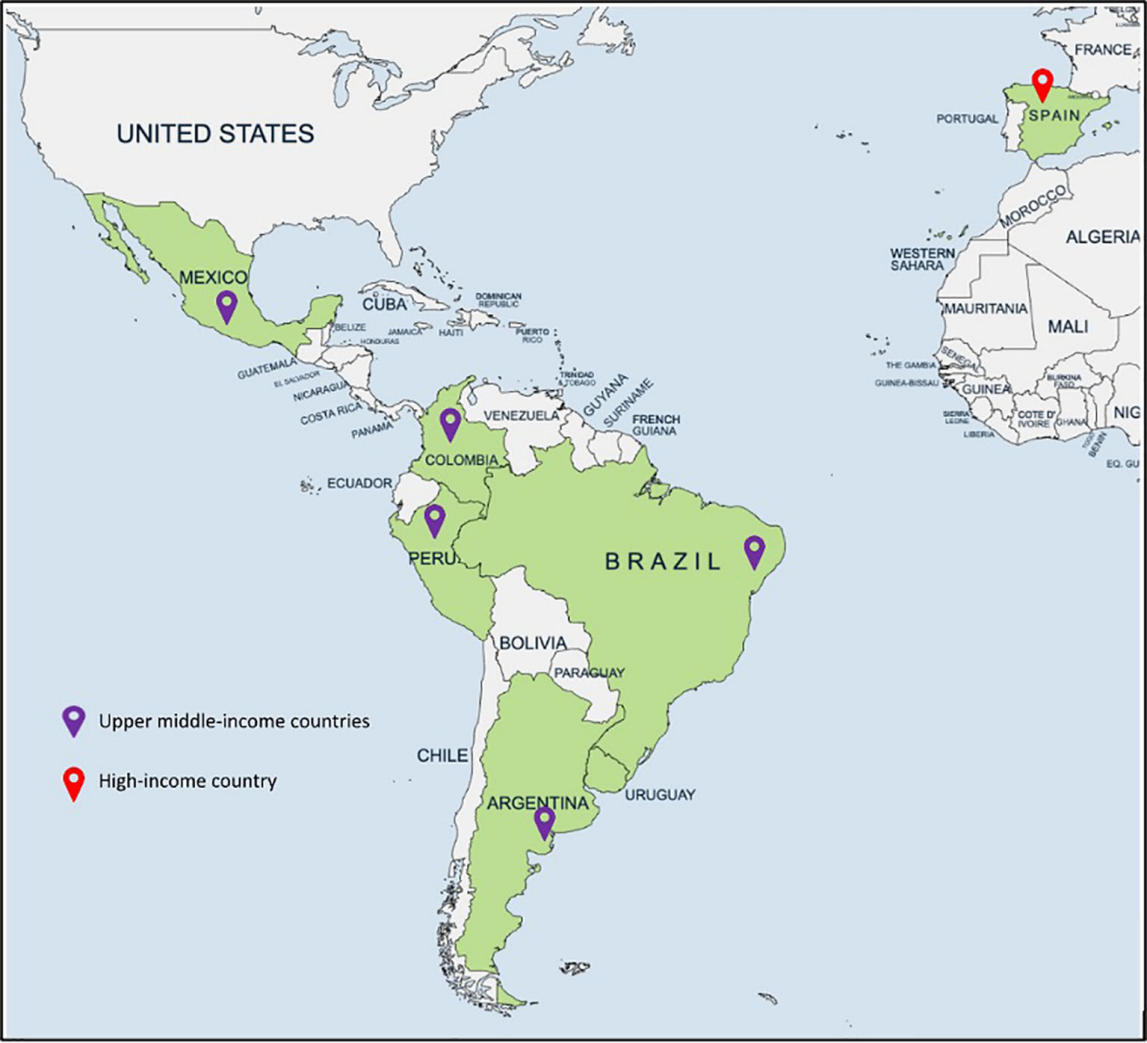
Map of participating EVAT Project centers by World Bank income level.

During the study period, six centers from five upper-middle-income countries in Latin America (Mexico, Peru, Colombia, Argentina, and Brazil) and one center from a high-income country (Spain) contributed pre- and post-PEWS implementation registry data for patients admitted to a transplant unit and were included in the analysis (**Table 1**, **Figure 1**). These centers reported an annual total of 168 HSCT; of these, 67% (*n =* 113) were allogeneic and 36% (*n =* 60) were haploidentical. HSCT was used in 68% (*n =* 114) of the cases for neoplastic diseases and received myeloablative conditioning in 49% (*n =* 82) of the procedures.

**Table 1:** Characteristics of the participating centers.

	Centers, *N* = 6	%/IQR
Upper middle-income countries^a^		
Argentina	1	16.7
Brazil	1	16.7
Colombia	1	16.7
Peru	1	16.7
Mexico	1	16.7
High-income country^a^		
Spain	1	16.7
Number of available pediatric HSCT beds		
3	2	33.3
6	2	33.3
8	2	33.3
Separate HSCT unit from PHO hospitalization ward		
Yes	4	66.7
No	2	33.3
Ward nursing ratios		
1 nurse/1 patient	1	16.7
1 nurse/2 patients	3	50.0
1 nurse/3 patients	2	33.3
Economic payment by patients		
No	6	100.0
Number of documented CDE	221	(3–12)
Total "by type of transplant"		
Annual number of autologous transplants	55	32.7
Annual number of allogeneic HLA-identical sibling transplants	29	17.3
Annual number of haploidentical transplants	60	35.7
Annual number of unrelated allogeneic transplants	24	14.3
CAR-T therapy		
Yes	1	14.3
No	5	85.7
Total CAR-T therapy		
Annual number of CAR-T therapy	8	100.0
Average percentage of transplant type in all centers		
Allogeneic transplantation for neoplastic diseases		68.2
Allogeneic transplantation for non-neoplastic diseases		15.2
Allogeneic transplants with myeloablative conditioning		48.6
Non-myeloablative transplants		34.7
Average percentage patients who required a second transplant		2.0

%, percentage; IQR, interquartile range; HSCT, hematopoietic stem cell transplantation; PHO, pediatric hematology oncology; CDE, clinical deterioration events; HLA, human leukocyte antigen; CAR-T, chimeric antigen receptor T-cell therapy.

a By World Bank income level.

### 3.2 CDE characteristics and outcomes

The centers reported a total of 221 CDE in 146 unique patients (one to six CDE/patient) during the study period. Of these, 71 events occurred among 50 patients pre-PEWS and 150 events occurred among 96 patients post-PEWS implementation; 39% CDE (*n =* 86/221) experienced more than one CDE. No patient experienced CDE both before and after PEWS implementation. Event characteristics are described in **Table 2** and **Supplementary Table S3**. Most events occurred in patients with hematologic malignancy (*n =* 153/221, 69%). CDE were attributable most often to sepsis or septic shock (*n =* 96, 43%) and respiratory distress (*n =* 62, 28%). The notable resource utilization and outcomes of the overall population included a median ICU length of-stay of 4.8 days [IQR 2.56–12.1 days] and a median duration of mechanical ventilation and vasoactive infusion of 3.2 days [IQR 1.1–10.8 days] and 1.9 days [IQR 0.9-4.3 days], respectively (**Supplementary Table S4**). Before transfer to a higher-care facility, ICU physicians evaluated the patient in 68% (*n =* 151/221) of the events, and 14% (*n =* 30/221) had ICU-level intervention on the ward. Ultimately, 98% (*n =* 216/221) were transferred to a higher level of care (ICU 97% [*n =* 210/216] and intermediate care unit 3% [*n =* 6/216]), with a median delay of 3.6 h [IQR 2.0–6.3 h] after CDE start. The outcomes of patients not transferred to a higher level of care are described in **Supplementary Table S5**. Overall, there were 39 CDE mortalities, representing an overall mortality rate of 18% (*n =* 39/221). One death occurred on the floor before transfer to the ICU; the remainder occurred in an ICU. Out of 39 deaths, 97.4% (*n =* 38/39) were attributable to treatment- or transplant-related mortality; only one was due to cancer progression or relapse.

**Table 2:** Characteristics of clinical deterioration events.

Characteristics	Variable	*n =* 221 *n* (%)
Patient characteristics		
Sex	Male	150 (68%)
	Female	70 (32%)
	Missing	1
Age	Years (median, IQR)	8.8 [4.3–14.2]
Oncologic diagnosis	Hematologic malignancy	153 (69%)
	Solid tumor	18 (8%)
	CNS tumor	4 (2%)
	Non-malignant hematology	45 (20%)
	Other	1 (0%)
Relapsed oncologic disease	Yes	55 (25%)
	No	166 (75%)
Event characteristics		
Reason for hospital admission	Initial diagnosis	8 (4%)
	Scheduled chemotherapy	6 (2%)
	HSCT	146 (66%)
	Disease relapse	8 (4%)
	Treatment of infection	31 (14%)
	Other non-infectious complication	6 (2%)
	Other	15 (7%)
	Missing	1
Primary clinical deterioration event	Unplanned ICU transfer	190 (86%)
	Vasoactive on ward	22 (10%)
	Mechanical ventilation on ward	6 (3%)
	CPR on ward	3 (1%)
	Ward death	0
Characteristics	Variable	(*N* = 195^a^)
Cause of deterioration^a^	Sepsis/septic shock	98 (50%)
	Respiratory distress/failure	63 (32%)
	Other cardiovascular dysfunction	51 (26%)
	Neurologic dysfunction	22 (11%)
	Hematologic dysfunction	10 (5%)
	Liver dysfunction	5 (3%)
	Renal dysfunction	4 (2%)
	Severe metabolic disturbance	3 (2%)
	Oncologic emergency (excluding lysis tumoral)	3 (2%)
	Other	12 (6%)
Ward ICU-level Intervention	Any (vasoactive, mechanical ventilation, CPR)	32 (14%)
	Vasoactive on ward	24 (11%)
	Mechanical ventilation on ward	5 (2%)
	CPR on ward	3 (1%)
	None	189 (86%)
Evaluated by ICU team on ward	Yes	151 (68%)
	No	70 (32%)
Transfer to a higher level-of-care (unit type)	No	5 (2%)
	ICU	210 (95%)
	IMCU	6 (3%)
Time from CDE start to ICU transfer	Hours (median, IQR)	3.6 [2.0–6.3]
Deterioration deaths	Yes	39 (18%)
	No	182 (82%)

CNS, central nervous system; HSCT, hematopoietic stem cell transplantation; ICU, intensive care unit; CPR, cardiopulmonary resuscitation; IQR, interquartile range; IMCU, intermediate care unit.

a Corresponds to “Select all that apply” in the survey; the sum of the count numbers may exceed the total number (*N*). The percentages are out of *N* given in the header.

### 3.3 CDE pre- and post-PEWS implementation

Events pre-PEWS had a similar distribution of age, sex, and status of oncologic disease as those post-PEWS (**Table 3**), though the distribution of oncologic diagnoses differed between these groups (*p = 0.*0495). Additionally, events occurring post-PEWS began earlier following hospital admission (median 22 [16–60] *vs*. 16 [11–28] days, *p =* 0.0016) and were less often due to respiratory distress or failure (38% [*n =* 27/71] *vs*. 23% [*n =* 35/150], *p =* 0.035).

**Table 3:** Comparisons of clinical deterioration events before and during/after PEWS implementation.

Variable	Options	Total *N* = 221	Pre-PEWS *N* = 71	During/after PEWS, *N* = 150	*p*-value^a^ (pre *vs*. during/after)
Patient characteristics					
Sex	Male	150 (68%)	53 (75%)	97 (65%)	0.2053
	Female	70 (32%)	18 (25%)	52 (35%)	
	Missing	1			
Age	Years (median, IQR)	8 [4–14]	8 [1–14]	9 [4–13]	0.3500
Relapsed oncologic disease	Yes	55 (25%)	13 (18%)	42 (28%)	0.1648
	No	166 (75%)	58 (82%)	108 (72%)	
Oncologic diagnosis	Hematologic Malignancy	153 (69%)	46 (65%)	107 (71%)	0.0495
	Solid tumor	18 (8%)	3 (4%)	15 (10%)	
	CNS tumor	4 (2%)	3 (4%)	1 (1%)	
	Non-malignant Hematologic	45 (20%)	18 (25%)	27 (18%)	
	Other	1 (0%)	1 (1%)	0 (0%)	
Deterioration event characteristics					
Days from hospital admission to event start		18 [11–34]	22 [15.5–60]	16 [11–28]	0.0016
Respiratory distress/failure as cause of deterioration	Yes	62 (28%)	27 (38%)	35 (23%)	0.0348
	No	159 (72%)	44 (62%)	115 (77%)	
Transfer to ICU	Yes No	216 (98%) 5 (2%)	70 (99%) 1 (1%)	146 (97%) 4 (3%)	1.0000
Any organ dysfunction at ICU transfer	No	89 (40%)	9 (13%)	80 (53%)	<0.0001
	Yes	132 (60%)	62 (87%)	70 (47%)	
Number of dysfunctional organs at ICU transfer, median (IQR)		1 [0–2]	2 [1–3]	0 [0–2]	<0.0001
PIM2 at time of ICU transfer		1.70 [1.20–6.20]	4.95 [1.4–7.9]	1.5 [1.2–3.38]	0.0006
	Missing	5			
CPR	Yes	7 (3%)	2 (3%)	5 (3%)	1.0000
	No	214 (97%)	69 (97%)	145 (97%)	
Resource utilization and outcome					
ICU length of stay	Days (median, IQR)	4.75 [2.6–12.1]	6 [2.1–14.3]	4.5 [2.7–10.8]	0.5889
	Missing	5			
Vasoactive infusions	Yes	110 (50%)	41 (58%)	69 (46%)	0.1371
	No	111 (50%)	30 (42%)	81 (54%)	
Mechanical ventilations	Yes	69 (31%)	28 (39%)	41 (27%)	0.0974
	No	152 (69%)	43 (61%)	109 (73%)	
CDE mortality	No	182 (82%)	54 (76%)	128 (85%)	0.1335
	Yes	39 (18%)	17 (24%)	22 (15%)	
Hospital mortality	No	155 (70%)	39 (55%)	116 (77%)	0.0012
	Yes	66 (30%)	32 (45%)	34 (23%)	

PEWS, pediatric early warning system; CNS, central nervous system; CDE, clinical deterioration event; ICU, intensive care unit; IQR, interquartile range; PIM2, Pediatric Index of Mortality 2; CPR, cardiopulmonary resuscitation.

a Two-sided *z*-test was used to compare incidence pre- and post-PEWS implementation within each characteristic.

Transfer to the ICU occurred at a lower severity of illness post-PEWS compared to the pre-PEWS period (**Table 3**). At the time of ICU transfer, events post-PEWS were less likely to have any organ dysfunction (87% [*n =* 62/71] pre *vs*. 47% [*n =* 70/150] post, *p <* 0.0001). The median number of organs with dysfunction at ICU transfer also decreased (2 [1–3] *vs*. 0 [0–2], *p <* 0.0001). Additionally, patients were transferred to the ICU earlier in their illness, with PIM2 scores decreasing post-PEWS implementation (median 5.0 [IQR 1.4–7.9] *vs*. 1.5 [IQR 1.2–3.4], *p < 0.*001).

ICU resource utilization, including the use of vasoactive infusions, mechanical ventilation, and ICU length-of-stay, was similar pre-PEWS and post-PEWS implementation (**Table 3**). There was a non-significant decrease in CDE mortality in the period after PEWS implementation (24% *vs*. 15%, *p* = 0.1335) and a significant decrease in hospital mortality (45% *vs*. 23%, *p =* 0.0012).

## 4 Discussion

In children hospitalized in six HSCT units involved in a quality improvement collaborative, we observed improved management of clinical deterioration events following implementation of PEWS, demonstrated by a lower severity of illness at transfer to a higher level of care, including lower median PIM-2 score and less organ dysfunction. This improvement occurred without an increase in ICU resource utilization, representing an overall improvement in the quality and safety of care for hospitalized HSCT patients without additional hospital costs. These findings are similar to data from the impact of PEWS on hospitalized children with cancer not undergoing HSCT (4, 5, 18).

Previously, there was concern about the performance of PEWS in children undergoing HSCT, as these tools were originally developed to identify clinical deterioration in hospitalized general pediatric patients (17). HSCT patients represent a unique and vulnerable group, facing higher mortality rates during critical illness and a greater need for escalated medical care (19). The increased vulnerability of HSCT recipients during critical illness stems from a complex interplay of pre-transplant factors (age, comorbid conditions (20), CMV seropositivity, the underlying disease and disease status), transplant-related factors (graft characteristics and source, intensity of the conditioning regimen, and immunosuppressive therapy), and post-transplant factors (time from HSCT to clinical deterioration, grade III or IV acute graft *vs*. host disease) (21, 22). Therefore, prompt detection of clinical deterioration is integral to prevent further complications and transplant-related mortality (19). As a result, in many hospitals, children undergoing HSCT are monitored more closely than pediatric oncology patients who are not undergoing HSCT. For example, the International Society of Pediatric Oncology (SIOP) Nursing Standards for Low- and Middle-Income Countries (LMICs) recommend lower nurse–patient ratios for transplant units compared to general oncology (23). Nonetheless, PEWS have been validated to predict deterioration among hospitalized children undergoing HSCT (3). Similarly, in this study, we observed earlier ICU transfer following PEWS implementation in six diverse hospitals providing HSCT care. The decision to transfer a patient to the ICU was based on each center’s customized PEWS response algorithm adapted to the local setting, a key element for successful implementation (24, 25). These findings add to existing evidence that a comprehensive system with standardized assessment and response such as PEWS, paired with a robust implementation strategy, can guide early action and improve hospital outcomes for these vulnerable patients (26).

In this study, we observed a decreased severity of illness on ICU transfer for hospitalized children undergoing HSCT, as measured by organ dysfunction and PIM2 scores at ICU transfers. Severity of illness at the time of initiation of critical care is strongly associated with poor outcomes and a common proxy for ICU mortality (16, 22). PEWS use is intended to detect deterioration promptly, thus expanding the time available to initiate interventions and prevent the development of irreversible organ dysfunction. Concordantly, early ICU evaluation and ICU transfer are recommended by recent international consensus to improve outcomes for hospitalized children undergoing HSCT (26). The observed decrease in organ dysfunction and PIM2 scores after PEWS implementation indicates that patients were transferred to the ICU earlier in the course of illness (13, 14). This suggests PEWS facilitates the identification, management, and triage of children undergoing HSCT, thus improving the quality and safety of care (9, 10, 17, 27, 28). In larger prior studies, this decrease in severity of illness was also shown to result in a reduction in CDE mortality. While we observed this trend in the current study, it did not reach statistical significance, possibly due to the smaller sample size (4).

The majority of deaths during clinical deterioration observed in this study were not due to cancer progression or relapse but rather due to treatment complications such as multi-organ dysfunction. This signifies potentially preventable transplant-related mortality that can be mitigated by improved supportive care. Existing evidence demonstrates that avoiding delays to the timely initiation of appropriate critical care and PICU-level interventions is an important strategy to decrease mortality in these patients (6, 17, 27, 29, 30). This study shows that the implementation of systems like PEWS that support nurses and doctors to perform a structured patient evaluation leads to earlier detection of CDE and earlier ICU transfer. In addition to systems that promote the early identification of clinical deterioration, advances in transplant procedures, the increasing use of immune effector cell therapies, candidate selection, and pediatric intensive care management (including prompt PICU and early critical care management strategies (31–36) are all strategies that improve clinical outcomes for children undergoing HSCT (26). However, support measures differ across centers and countries, highlighting the need for further research to alleviate treatment-related mortality while considering local contexts.

Our study has several limitations. The study design, which compares CDE occurring pre-PEWS and post-PEWS implementation, weakens the causal inference regarding the impact of PEWS implementation on the observed decrease in the severity of CDE. Nevertheless, the general characteristics of the CDE were similar pre-PEWS and post-PEWS implementation, and the PIM2 score is a robust objective predictor of mortality risk and time of initiation of critical care (16). Our sample size was limited due to the number of pediatric HSCTs performed at Proyecto EVAT centers that completed PEWS implementation. This may have restricted our ability to detect smaller effects related to PEWS implementation, including the impact on CDE mortality. Additionally, due to the limited number of events pre- and post-PEWS implementation, the results were reported at the event-level only, and controlling for multiple sampling was not possible statistically. A larger-scale trial will be needed to confirm these results through patient-level analyses, such as using generalized estimating equations. Another limitation was the manner by which children receiving HSCT were identified in the Proyecto EVAT CDE registry used in this study. This registry was initially designed for patients diagnosed with cancer and hospitalized in centers mostly without access to HSCT. Therefore, data on the type of transplant, date of transplant, conditioning intensity, donor match, cell processing, graft versus host, and diagnostic criteria for transplant indication, among other factors, were not collected in the registry. For this reason, patients undergoing HSCT were identified by their unit of hospitalization. It is possible that patients with a history of remote HSCT were thus missed in this analysis. Additionally, the lack of details on transplant-related factors prevents us from controlling for these factors in our analysis. Finally, prior studies have highlighted additional benefits of PEWS implementation in the care of children with cancer, including enhanced staff empowerment, improved interdisciplinary communication, higher perceived quality of care, and cost savings, which were not measured in this research (18, 27, 37). Future work should investigate the impact of PEWS on these multi-level outcomes for children undergoing HSCT.

## 5 Conclusion

This study suggests that PEWS implementation across six centers with diverse HSCT capabilities in Latin America and Spain improved hospital outcomes for children undergoing HSCT by facilitating the early detection of critical illness and prompt ICU transfer at a lower severity of illness. These data contribute to the current literature supporting the effectiveness of PEWS in improving care for pediatric oncology patients, including those undergoing HSCT.

## Data Availability

The raw data supporting the conclusions of this article will be made available by the authors, without undue reservation.
