# Supplementary material for: Impact of implementing a pediatric early warning system on outcomes in hematopoietic stem cell transplant units in South America and Europe
Source: Front Oncol. 2025 Dec 9;15:1712611. doi: 10.3389/fonc.2025.1712611 (PMC12722999; doi:10.3389/fonc.2025.1712611)
Supplement: Supplementary file 1 [file DataSheet1.docx]

Supplementary Material

**Appendix: Supplementary Tables and Figures**

| **Appendix** | **Page** |
| --- | --- |
| Supplementary Figure 1. PEWS (EVAT) Scoring Tool | 2 |
| Supplementary Figure 2. PEWS (EVAT) Sample Action Algorithm | 3 |
| Supplementary Figure 3. English-Language Case Report Form | 4 |
| Supplementary Table 1. Participating Proyecto EVAT Center Details | 6 |
| Supplementary Table 2. Evaluated the level of the Event, Patient, and Hospital | 7 |
| Supplementary Table 3: Description of clinical deterioration events | 10 |
| Supplementary Table 4. Duration of clinical deterioration events and resource utilization | 13 |
| Supplementary Table 5. Clinical Deterioration Events not Transferred to a Higher Level-of-Care | 14 |
| Supplementary Table 6. Authorship Group: EVAT HSCT Study Group | 15 |

**Supplementary Figure 1. PEWS (EVAT) Scoring Tool**

**
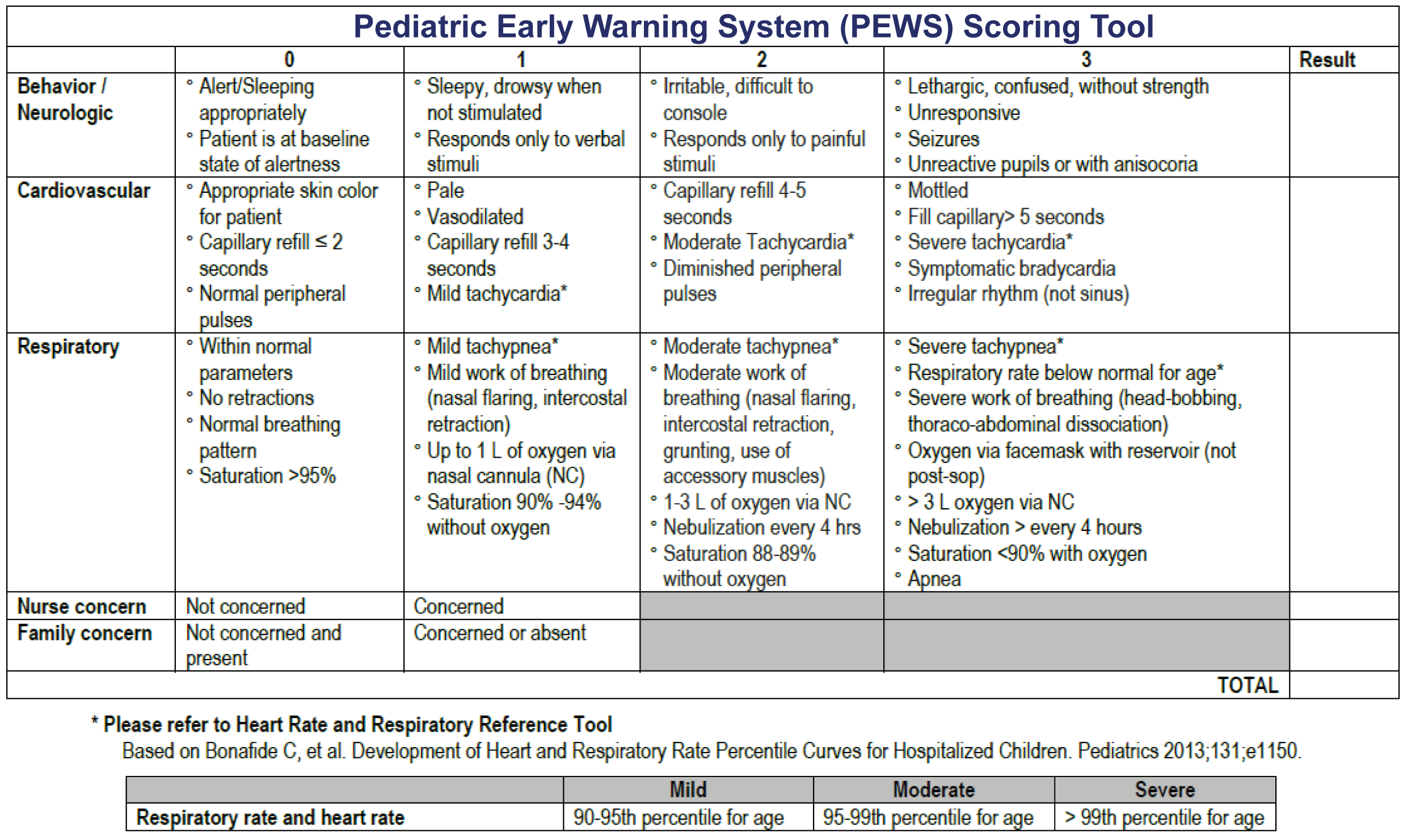
**

**Supplementary Figure 2. PEWS (EVAT) Sample Action Algorithm**


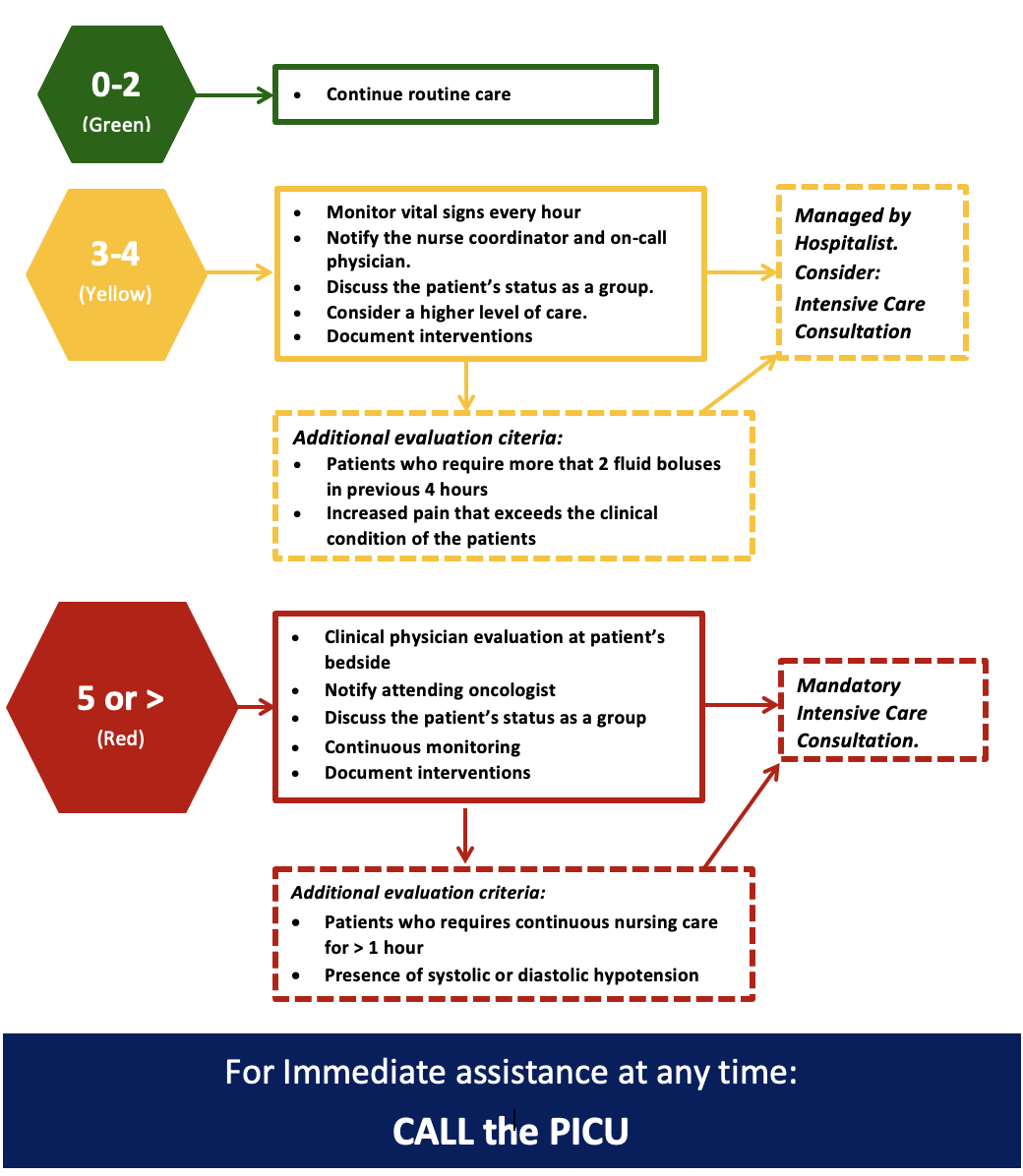


**Supplementary Figure 3. English-Language Case Report Form
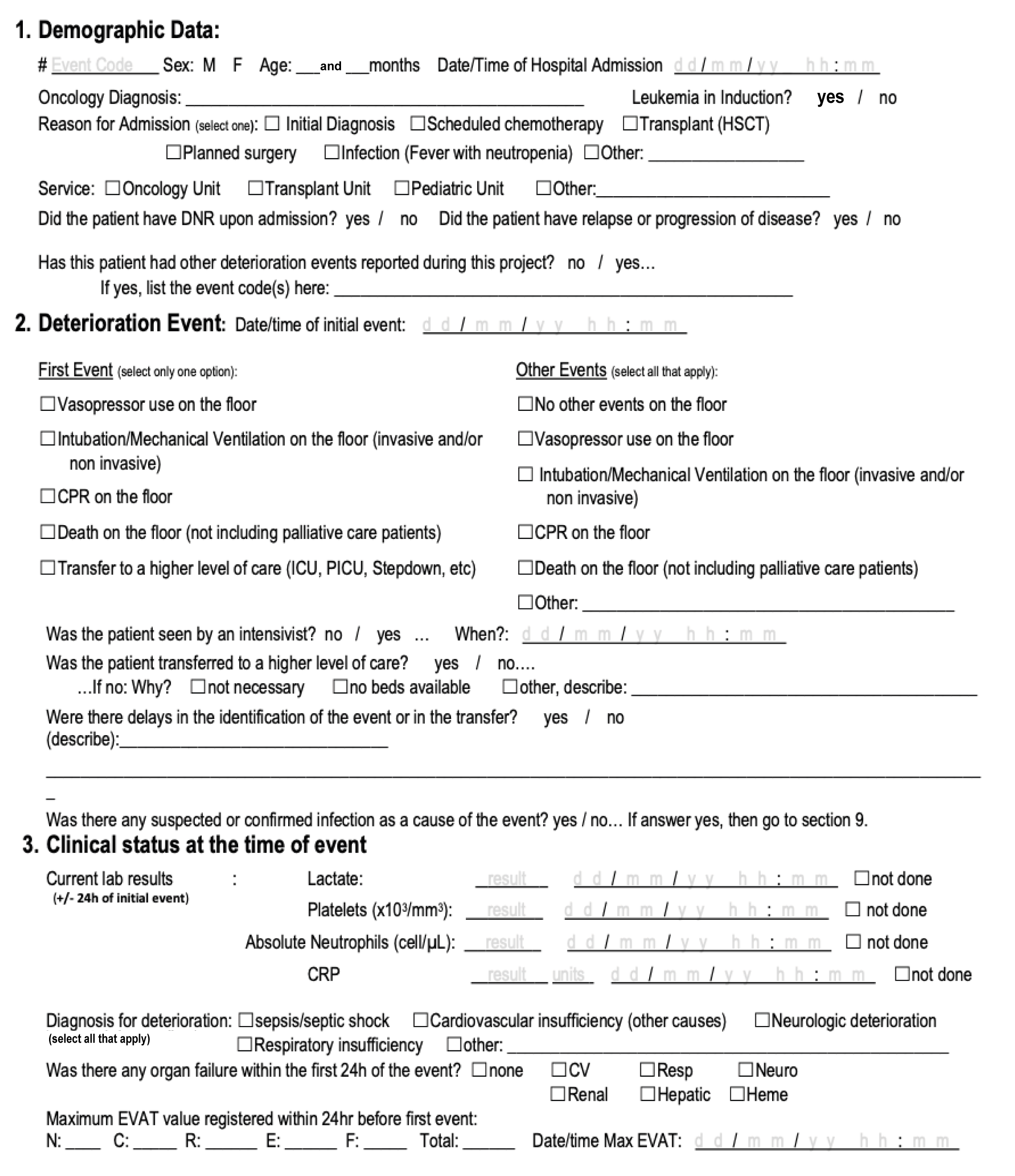
**

**
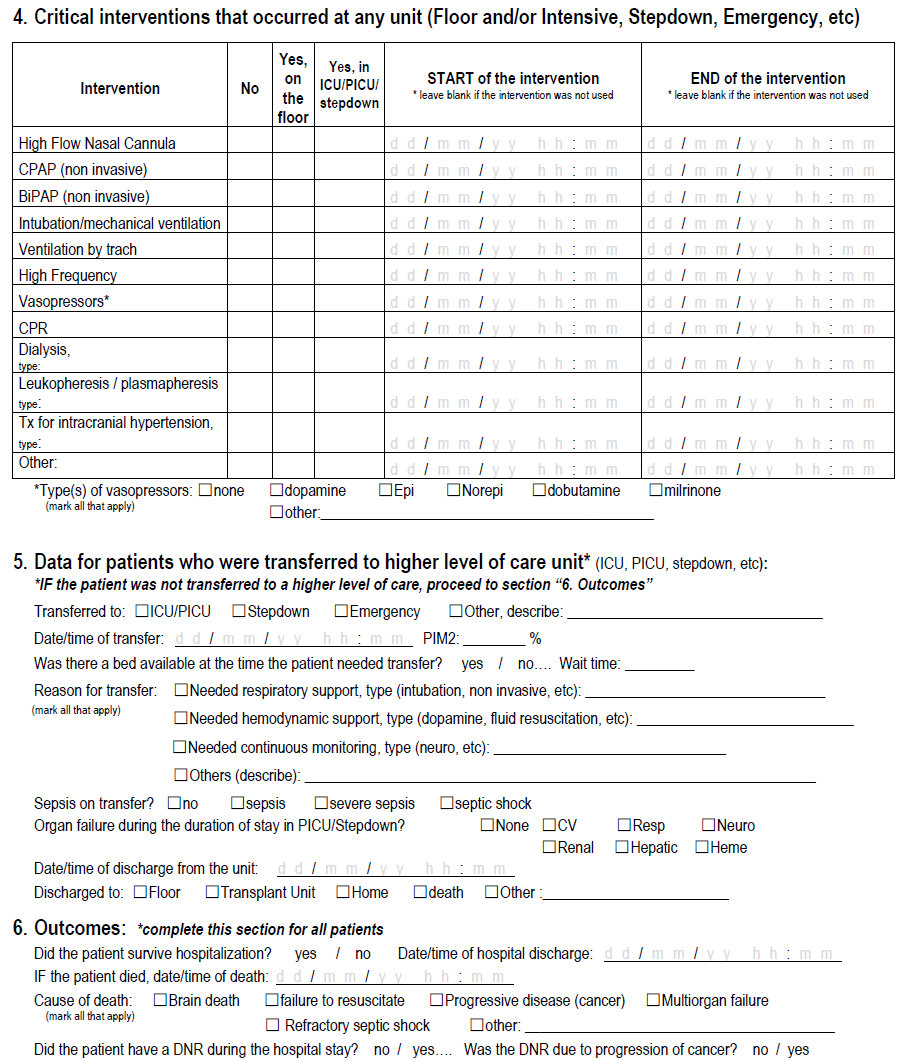
**

**Supplementary Table 1. Participating Proyecto EVAT Center Details**

| **Country** | **City** | **Center** | **Date of pre-PEWS^a^** | **Date to PEWS implementation** | **Date of end of data collection^b^** |
| --- | --- | --- | --- | --- | --- |
| Argentina | Buenos Aires | Hospital Universitario Austral | 20/04/2019 | 1/06/2020 | 30/11/2021 |
| Brazil | Barretos | Hospital do Amor/ Hospital de Cancer Infanto Juvenil de Barretos | 15/08/2020 | 14/03/2022 | 30/03/2024 |
| Colombia | Cali | Clinica Imbanaco | 4/04/2019 | 19/02/2020 | 31/03/2022 |
| Peru | Lima | Instituto Nacional de Enfermedades Neoplásicas (INEN) | 11/04/2017 | 07/05/2018 | 31/08/2020 |
| Mexico | Guadalajara | Hospital Civil de Guadalajara | 1/02/2018 | 08/04/2019 | 31/07/2021 |
| Spain | Madrid | Hospital Universitario La Paz | 1/07/2020 | 27/09/2021 | 30/04/2024 |

^a^(Date of start of tracking)

^b^(If 18m post collected). If empty= data collection ongoing

Dates are presented in format of day/month/year

**Supplementary Table 2. Evaluated the level of the Event, Patient, and Hospital**

| **Level** | **Risk Factor** | **Variable Description** | **Notes** |
| --- | --- | --- | --- |
| Event (all) | Primary event | Unplanned ICU transfer; vasoactive medications on ward; mechanical ventilation on ward; CPR on ward; non-palliative ward death | Event that first met criteria for CDE |
|  | Reason for hospital admission | New or relapsed cancer; scheduled admission; acute (unscheduled) admission; other |  |
|  | Hospital service at start of deterioration | Oncology ward, Pediatric ward, Oncology-intermediate ward (UNOP only), Transplant ward, other |  |
|  | Any ICU-level interventions on ward | Yes/No |  |
|  | Ward cardiopulmonary arrest | Yes/No |  |
|  | Ward cardiac arrest | Yes/No |  |
|  | Evaluated by ICU on ward | Yes/No |  |
|  | Transferred to higher-level-of-care (ICU or other) | Yes/No |  |
|  | Documented PEWS | Yes/No |  |
|  | Lactate | Continuous; mmol/L ( | Collected within 24 hours of the start of the CDE |
|  | Absolute neutrophil count (ANC) | Continuous; cells/μL | Collected within 24 hours of the start of the CDE |
|  | Platelet count | Continuous; x10^3^/mm | Collected within 24 hours of the start of the CDE |
|  | C-Reactive Protein (CRP) | Continuous; mg/L | Collected within 24 hours of the start of the CDE |
|  | Delay in identification of deterioration or ICU transfer | Yes/No | As categorized by clinical team |
|  | First CDE | Yes/No | Yes: patients first CDE, No: patient has had prior CDEs |
|  | Time (shift) during event start | Day Shift  Night Shift |  |
|  | Day during event start | Weekday: Monday-Friday  Weekend: Saturday or Sunday |  |
|  | Reason for deterioration | Sepsis/septic shock; other cardiovascular dysfunction; neurologic; respiratory insufficiency/failure; pancreatitis; metabolic derangement; tumor lysis syndrome; renal disfunction; hepatic disfunction; other: oncologic emergency; hematologic dysfunction; other | Choose all |
|  | Organ dysfunction | Yes/No dysfunction of CV, respiratory, neurologic, renal, hepatic, hematologic organs, and total number of organs with disfunction | Defined as per Goldstein et al; evaluated at 1) start of event and 2) time of transfer to a higher level-of-care (only among patients transferred) |
| Event (among CDE transferred to a higher level-of-care | PIM2 | Continuous | Calculated as per Slater *A et al*, (2003), using data on admission to higher level-of-care |
|  | ICU bed availability at time of transfer | Yes/No; hours in delay from need for ICU-to-ICU transfer for those with “No” |  |
|  | Sepsis at ICU transfer | No; Sepsis; Severe Sepsis; Septic Shock | As per Goldstein et al |
|  | Higher level-of-care | ICU; intermediate care unit; emergency room; COVID area; other |  |
|  | Reason for ICU transfer | Respiratory support; hemodynamic support; monitoring; other | Choose all |
| Patient | Age | Continuous, in years |  |
|  | Sex | Male or Female |  |
|  | Oncologic Diagnosis | Hematologic malignancy; non-CNS solid tumor; CNS solid tumor; non-malignant hematology; other |  |
|  | Relapsed oncologic disease | Yes/No |  |
| Hospital | Country Income Level^a^ | HIC, UMIC, LMIC | Defined by World Bank Criteria |
|  | Hospital Governance | Private; Public; Mixed (Public + Private) |  |
|  | Hospital Type | Pediatric Oncology; Pediatric Multidisciplinary; Oncology (adult and pediatric); General (adult and pediatric); Women’s’ and Children’s’ |  |
|  | Separate PHO unit | Yes/No |  |
|  | Type of ICU | PHO ICU; Pediatric ICU; Adult ICU (accepting pediatric patients); no ICU |  |
|  | Hospital Department of Quality and Safety | Yes/No |  |
| HSCT Unit | Number of pediatric beds available in the HSCT unit | continuous |  |
|  | Separate HSCT unit | Yes/No |  |
|  | Ward nurse:patient ratio | Average # patients per ward nurse |  |
|  | Financial payment of HSCT by patients | Yes/No |  |
|  | Annual number of autologous transplants | continuous |  |
|  | Annual number of allogeneic HLA-identical sibling transplants | continuous |  |
|  | Annual number of haploidentical transplants | continuous |  |
|  | Annual number of unrelated allogeneic transplants | continuous |  |
|  | Annual number of CAR-T therapies | continuous |  |
|  | Percentage of allogeneic transplants due to neoplastic diseases | Average # allogeneic transplants per neoplastic diseases |  |
|  | Percentage of allogeneic transplants due to non-neoplastic diseases | Average # allogeneic transplants per non-neoplastic diseases |  |
|  | Percentage of allogeneic transplants with myeloablative conditioning | Average # allogeneic transplants per myeloablative conditioning |  |
|  | Percentage of non-myeloablative transplants | Average # transplants per non-myeloablative conditioning |  |
|  | Number of inpatient beds | continuous |  |
|  | Average number of annual new pediatric cancer patients | continuous |  |
|  | Ward nurse:patient ratio | Average # patients per ward nurse |  |

**Abbreviation**s: **ICU**=Intensive Care Unit. **CPR**=Cardiopulmonary Resuscitation. **CDE**=Clinical Deterioration Event. **CNS**=Central Nervous System. **^a^Country Income Level** by World Bank income level; **UMIC**=Upper Middle-Income Countries; **HIC=**High-Income Countries; **LMIC=**Low and Middle-Income Countries. **PHO**=Pediatric Hemato-Oncology. **HSCT**=Hematopoietic Stem Cell Transplantation. **HLA=**Human Leukocyte Antigen. **CAR-T**=Chimeric Antigen Receptor T-Cell Therapy.

**Supplementary Table 3: Description of clinical deterioration events**

| **Characteristics** | **N (%) / Median (IQR)**  **(Total=221)** |
| --- | --- |
| **Deterioration Deaths** | N=39 |
| Yes | 39 (18%) |
| No | 182 (82%) |
| **Deterioration death by transfer to ICU** |  |
| Yes transfer to ICU | 38 (18%) |
| No transfer to ICU | 1 (2%) |
| **Deterioration death by mechanical ventilation (invasive vs non-invasive)** |  |
| Yes mechanical ventilation | 35 (59%) |
| No mechanical ventilation | 4 (2%) |
| **Deterioration death by mechanical ventilation (non-invasive; CPAP or BiPAP)** |  |
| Yes non-invasive (CPAP or BiPAP) | 14 (45%) |
| No non-invasive (CPAP or BiPAP) | 25 (13%) |
| **Deterioration death by vasoactive infusion** |  |
| Yes vasoactive infusion | 35 (32%) |
| No vasoactive infusion | 4 (4%) |
| **Deterioration death by CPR** |  |
| Yes CPR | 12 (92%) |
| No CPR | 27 (13%) |
| **Deterioration death by renal replacement** |  |
| Yes renal replacement | 15 (62%) |
| No renal replacement | 24 (12%) |
| **Location of Deterioration Deaths** |  |
| HSCT unit prior to ICU transfer | 1 (3%) |
| ICU | 38 (97%) |
| **ICU interventions on the HSCT unit** | N=221 |
| Yes | 30 (14%) |
| No | 191 (86%) |
| **HSCT unit Cardiopulmonary Arrest** |  |
| Yes | 7 (3%) |
| No | 214 (97%) |
| **HSCT unit and ICU vasoactive infusions** |  |
| Yes | 110 (50%) |
| No | 111 (50%) |
| **HSCT unit and ICU CPR** |  |
| Yes | 13 (6%) |
| No | 208 (94%) |
| **HSCT unit and ICU Renal Replacement** |  |
| Yes | 24 (11%) |
| No | 197 (89%) |
| **Reason for Hospital Admission** |  |
| Scheduled Chemotherapy | 6 (3%) |
| Other non-infectious acute complications | 6 (66%) |
| Initial Diagnosis | 8 (4%) |
| Relapse | 8 (4%) |
| Other | 15 (7%) |
| Treatment of infection | 31 (14%) |
| HSCT | 146 (66%) |
| Missing | 1 |
| **Relapse or progression of oncological diagnosis** |  |
| Yes | 55 (25%) |
| No | 166 (75%) |
| **Previous CDE** |  |
| Yes | 86 (39%) |
| No | 135 (61%) |
| **CDE shift** |  |
| Day shift | 96 (43%) |
| Night shift | 125 (57%) |
| **CDE primary event on the HSCT unit** |  |
| Mortality | 0 (0%) |
| CPR | 3 (1%) |
| Mechanical Ventilation (invasive and/or non-invasive) | 6 (3%) |
| Vasopressors | 22 (10%) |
| Transfer to a high-care unit (intensive care, emergency, intermediate, etc.) | 190 (86%) |
| **Intensive care assessment before transfer to ICU** |  |
| Yes | 216 (98%) |
| No | 5 (2%) |
| **Diagnosis of deterioration*** |  |
| Sepsis/septic shock | 98 (50%) |
| Cardiovascular dysfunction (other causes) | 51 (26%) |
| Neurological deterioration | 22 (11%) |
| Respiratory distress/failure | 63 (32%) |
| Pancreatitis | 0 |
| Severe metabolic disturbance | 3 (2%) |
| Tumor lysis syndrome | 0 |
| Renal dysfunction | 4 (2%) |
| Liver dysfunction | 5 (3%) |
| Oncologic emergency (excluding tumor lysis) | 3 (2%) |
| Hematologic dysfunction (anemia, coagulopathy, or hemorrhage) | 10 (5%) |
| Other | 12 (6%) |
| Total | 271 |
| **Number of organs with failure** |  |
| 0 | 27 (12%) |
| 1 | 74 (33%) |
| 2 | 73 (33%) |
| 3 | 27 (12%) |
| 4 | 10 (5%) |
| 5 | 10 (5%) |
| **Death diagnosis*** | (N=39) |
| Multi-organ dysfunction | 31 (79%) |
| Refractory septic shock | 11 (28%) |
| Failure to resuscitate | 8 (21%) |
| Cancer progression | 1 (3%) |
| Other | 3 (8%) |

**Abbreviations: ^a^Deterioration Death**=considered if a patient is transferred to ICU and died in ICU, or if died after ICU discharge and difference between is <=24 hrs, or if primary event or other event is death (i.e., death happened on the floor). **IQR**=Interquartile Range.  **CDE**=Clinical Deterioration Event. **ICU**=Intensive Care Unit. **CPR**=Cardiopulmonary Resuscitation. **HSCT**=Hematopoietic Stem Cell Transplantation. **^b^**Correspond to 'Select all that apply’ in the survey. The sum of count numbers may exceed total number (N).

**Supplementary Table 4. Duration of clinical deterioration events and resource utilization**

| **Variable** | **N. Obs** | **N. Missing** | **N** | **Min** | **Q25** | **Median** | **Q75** | **Max** | **Mean** | **SD** |
| --- | --- | --- | --- | --- | --- | --- | --- | --- | --- | --- |
| Event duration^a^ | 221 | 1 | 220 | 0.04 | 2.6 | 4.7 | 12.0 | 61.9 | 9.3 | 10.9 |
| ICU length of stay^b^ (in days) | 216 | 1 | 215 | 0.11 | 2.6 | 4.8 | 12.1 | 61.9 | 9.4 | 10.9 |
| Hospital length of stay^c^ (in days) | 154 | 0 | 154 | 1.81 | 26.8 | 38.1 | 65.9 | 239.2 | 55.9 | 47.8 |
| Time to event start (in days) | 221 | 1 | 220 | 0.02 | 11.1 | 18.3 | 34.1 | 191.4 | 29.4 | 32.1 |
| Mechanical ventilation duration (in days) | 69 | 0 | 69 | -Inf | 1.1 | 3.2 | 10.8 | 60.7 | -Inf | NaN |
| Invasive mechanical ventilation duration (in days) | 59 | 0 | 59 | 0.00 | 1.2 | 5.1 | 15.8 | 60.7 | 10.3 | 13.4 |
| Vasoactive infusion duration (in days) | 110 | 0 | 110 | 0.00 | 0.9 | 1.9 | 4.3 | 47.5 | 4.9 | 8.1 |
| Number of hours from CDE start to transfer time to ICU | 26 | 0 | 26 | 0.00 | 2.0 | 3.6 | 6.3 | 71.7 | 9.5 | 16.6 |
| Number of hours to bed wait | 216 | 144 | 72 | 0.00 | 0.00 | 2.00 | 4.00 | 48.00 | 3.03 | 5.90 |
| PIM2 (%) | 216 | 0 | 216 | 0.20 | 1.2 | 1.7 | 6.2 | 94.6 | 5.1 | 10.1 |

**Abbreviations:** **Obs**=observations. **CDE**=Clinical deterioration events. **^a^Event duration**= if the outcome of an event is deterioration death, then the event duration is the time between event start to death time. If the event indicated that the patient was transferred to the ICU, the event duration is the time between event start to ICU discharge. If the event indicated that the patient wasn’t transferred to the ICU and at least one ICU intervention happened on the floor, the event duration is the time between event start to last time of intervention on the floor. **ICU**=-intensive care unit. **PIM2**=Pediatric Index of Mortality 2. **^b^ICU length of stay**= is calculated for all the patients who were transferred to the ICU. This also includes the patients who had interventions on the floor and were later transferred to the ICU. **ICU stay days**= ICU Discharge Date - ICU Admission Date. **^c^Hospital length of stay**= Hospital Discharge Date - Hospital Admission Date.

**Supplementary Table 5: Clinical Deterioration Events not Transferred to a Higher Level-of-Care**

| **Event Number** | **Pre or Post PEWS** | **Definition of CDE** | **Outcome** |
| --- | --- | --- | --- |
| 1 | Pre-PEWS | Vasopressors on ward | Survived this event, died in ICU during second CDE, also occurring pre-PEWS implementation |
| 2 | Post-PEWS | CPAP and vasopressors on ward | Survived, no other CDEs |
| 3 | Post-PEWS | Vasopressors on ward | Survived, no other CDEs |
| 4 | Post-PEWS | Vasopressors on ward | Survived, no other CDEs |
| 5 | Post-PEWS | CPR and intubation/ mechanical ventilation on ward | Died |

**Abbreviations: PEWS=**Pediatric Early Warning System. **CDE**=Clinical deterioration events. **ICU**=-intensive care unit. **CPAP=** mechanical ventilation non-invasive. **CPR**=Cardiopulmonary Resuscitation.

**Supplementary Table 6. Authorship Group: EVAT Study Group**

| **First Name / Middle Initial** | **Last Name** | **Degree** | **Institution** | **Location (city, country)** |
| --- | --- | --- | --- | --- |
| Monica L. | Quijano-Lievano | MsN | Clinica Imbanaco, Quiron Salud | Cali, Colombia |
| Oscar | Ramirez | MD | Clinica Imbanaco, Quiron Salud | Cali, Colombia |
| **Yichen** | Chen | PhD | St. Jude Children's Research Hospital | Memphis, United States |
| Maricela | **Robles-Murguia** | MS, MSM | St. Jude Children's Research Hospital | Memphis, United States |
| **Hilmarie** | **Muñiz-Talavera** | PhD | St. Jude Children's Research Hospital | Memphis, United States |
| Adolfo | **Cárdenas-Aguirre** | MD | St. Jude Children's Research Hospital | Memphis, United States |
| Carlos | **Portilla** | MD | Clinica Imbanaco, Quiron Salud | Cali, Colombia |
| Diana | **Castrillon** | MD | Clinica Imbanaco, Quiron Salud | Cali, Colombia |
| Diana | Rendon | MD | Clinica Imbanaco, Quiron Salud | Cali, Colombia |
| **Andreia** | **Ribeiro Pereira Aguiar De Paula** | MD | **Hospital do Amor/ Hospital de Cancer Infanto Juvenil de Barretos** | **Barretos, Brazil** |
| **Rosdali** | **Diaz-Coronado** | MD | **Instituto Nacional de Enfermedades Neoplásicas (INEN)** | **Lima, Perú** |
| Maria | Sánchez-Martín | MD | **Hospital Universitario La Paz** | **Madrid, España** |
| Silvio | Torres | MD | **Hospital Universitario Austral** | **Buenos Aires, Argentina** |
| Veronica | Soto Chávez | MD | **Hospital Civil de Guadalajara** | **Guadalajara, México** |
| Meenakshi | Devidas | PhD | St. Jude Children's Research Hospital | Memphis, United States |
| Asya | Agulnik | MD | St. Jude Children's Research Hospital | Memphis, United States |
